# Dynamic assessment of the ecological value of cultivated land based on the Gompertz curve model: A case study of Lezhi County, China

**DOI:** 10.1371/journal.pone.0339281

**Published:** 2025-12-29

**Authors:** Li Yuan, Hongjie Chen, Jing Xu, Haidong Wang

**Affiliations:** 1 School of Agricultural and Forestry Economics and Management, Lanzhou University of Finance and Economics, Lanzhou, China; 2 Belt and Road Economic Research Institute, Lanzhou University of Finance and Economics, Lanzhou, China; 3 School of Statistics and Data Science, Lanzhou University of Finance and Economics, Lanzhou, China; Peking University, CHINA

## Abstract

With the continuous advancement of ecological compensation mechanisms, there is an urgent need to refine the dynamic evaluation of the ecological value of cultivated land (EVCL). This study constructs a dynamic assessment model for the EVCL based on the Gompertz curve, selecting Lezhi County in Sichuan Province, China, as the study area. The impact of the evolution of ecological compensation policies on the EVCL is examined. Using the functional value method, the study quantifies four key ecosystem services: agricultural product supply, water conservation, carbon sequestration and oxygen release, and soil and water conservation. It estimates that the ecological value per hectare of cultivated land reached CNY 704,250 in 2022, expressed in constant 1990 prices using the official GDP deflator published by the National Bureau of Statistics of China. A Gompertz curve linear equation was developed through logarithmic linearization, and parameter estimation was supported by the GDP growth rate as a proxy variable. This approach ensured statistical consistency and avoided overfitting. The model was compared with the traditional Pearl curve using quantitative indicators such as the coefficient of determination (R²) and root mean square error (RMSE). These measures highlight the superior correction capacity and fitting stability of the Gompertz model. To ensure temporal consistency, all monetary values were harmonized by converting nominal series into constant 1990 prices before integration with historical data (1979–2022) and GM(1,1) forecasts up to 2052. This integration provided a coherent time series for calculating development stage coefficients and projecting EVCL. The results indicate that the ecological value follows an asymmetric S-shaped growth trajectory as development stages advance, aligning with the gradual evolution of ecological compensation policies and increasing institutional maturity. By linking quantitative model validation with policy evolution, the study not only estimates the EVCL from 1979 to 2052 but also provides a methodological reference for evaluating ecological compensation policies across regions.

## 1. Introduction

Cultivated land serves as the foundation for food production and is a critical resource for maintaining ecological security [1]. However, with the rapid advancement of agricultural modernization and urbanization, the ecosystem services provided by cultivated land are increasingly threatened by degradation, highlighting the urgent need for effective evaluation and compensation mechanisms. Traditional approaches to assessing the ecological value of cultivated land (EVCL) have largely focused on static, single-period estimations, lacking a comprehensive depiction of the dynamic evolution of ecological value [2,3]. Consequently, these methods fail to fully capture the impacts of institutional supply and ecological compensation policies, limiting their applicability for long-term planning and policy evaluation. Internationally, policy frameworks that promote ecological compensation and sustainable land management emphasize the need for long-term monitoring and adaptive mechanisms. These frameworks highlight the importance of linking ecological value assessment to institutional design, providing a broader context for dynamic evaluation. In the context of accelerating economic growth, urbanization, and the continual refinement of ecological compensation policies, existing quantitative models struggle to accurately represent the long-term dynamic changes in ecological value.

The Gompertz curve, characterized by its asymmetric S-shaped growth pattern, has been widely applied in areas such as biological growth, technology diffusion, and policy evolution [4–7]. Its non-symmetrical developmental trajectory also aligns with the way institutional and policy interventions evolve, starting slowly, accelerating during expansion, and stabilizing as policies mature. This makes it particularly suitable for modeling the policy-driven adjustment of ecological value. Compared with traditional symmetric models, the Gompertz-based framework allows a closer integration of ecological value assessment with policy evolution, thereby providing a more realistic reflection of institutional influence. Based on the Gompertz curve, this study proposes a new approach for dynamically assessing the EVCL, aiming to solve the problem of dynamic adjustment and phase change of ecological value under institutional provisioning. Compared to traditional models, constructing a development stage coefficient model using the Gompertz curve enables the adjustment of the amount of ecological value of cropland and facilitates the evaluation of ecological compensation effectiveness over time [8,9].

Lezhi County, located in the central Sichuan Basin and in the upper reaches of the Yangtze River, possesses rich ecological functions and a strong foundation in agricultural production. Since its designation in 1992 as one of China’s pilot areas for “Sustainable Agriculture and Rural Development Research,” Lezhi County has accumulated extensive practical experience in ecological agricultural development. Its combination of long-term ecological practices, rapid socioeconomic change, and its role as an early pilot region makes it a representative case for examining the dynamic interaction between cultivated land value and institutional supply. This dual significance—ecological and institutional—highlights the suitability of Lezhi County as a case study. By embedding the analysis within a broader policy and institutional framework, the study responds to international discussions on how ecological compensation mechanisms can adapt to socio-economic transitions.

By constructing a development stage coefficient model to measure the EVCL, it can reveal the impact of ecological compensation policies on the realization of EVCL, and provide a scientific basis for the formulation of ecological compensation policies in China and other countries and regions [10]. This approach addresses the limitation of static evaluation methods by capturing how policy evolution dynamically shapes ecological value. It therefore provides both methodological guidance and a practical reference for regions undergoing similar institutional transitions.

## 2. Literature review

### 2.1. Demand-side approaches and Pearl’s curve

In the field of economics, early empirical research on development stage coefficients originated from economic growth theory. Simon Kuznets conducted one of the earliest empirical analyses, utilizing GDP data to investigate changes in income distribution across different stages of development [11]. Building on this foundation, Barro and Sala-i-Martin refined the understanding of development stage coefficients through cross-country regression models [12]. In more recent years, modern methodologies have integrated hidden Markov models [13] and Bayesian structural equation models [14], providing a more nuanced quantification of development stage transitions within complex systems and enriching the empirical foundations for development stage coefficient analysis.

In the domain of resource and environmental value assessment, Li Jinchang introduced the Pearl S-curve from biology into environmental valuation, constructing a quantitative model of willingness to pay (WTP) relative to development stages [15]. Subsequent research incorporated marginal utility theory into the calculation of development stage coefficients, thereby strengthening the theoretical underpinnings of the model [16]. As the field progressed, behavioral economics perspectives were integrated, considering social-psychological factors such as environmental cognition and institutional trust to enhance the explanatory power of the models [10].

Early methodologies for estimating development stage coefficients primarily relied on the contingent valuation method (CVM), establishing a normative framework for measuring WTP [17]. The later incorporation of panel data analysis revealed the nonlinear characteristics of WTP changes across different development stage [18]. More recently, spatial econometric approaches have been employed, providing methodological support for the formulation of regionally differentiated policies [19,20].

Existing studies can be grouped into three major streams. The first focuses on demand-side dynamics, using Pearl’s symmetric S-curve to describe how WTP evolves with income growth and public awareness of ecosystem services. The second emphasizes methodological innovation, introducing tools such as CVM, spatial econometrics, and long-term monitoring to improve coefficient estimation. The third explores supply-side factors, though this stream remains limited and often underdeveloped. Such categorization helps clarify both the achievements and the remaining gaps in the literature.

### 2.2. Methodological developments

The application of development stage coefficients has evolved from macro to micro scales and from static to dynamic perspectives. Spatially, research has shifted from the national scale [21,22] to watershed [23] and community levels [24]. Temporally, the analytical perspective has moved from static cross-sectional analyses to dynamic tracking studies, exemplified by Zou et al. [25] and Ma et al. [26]. Recent research has addressed coefficient adjustments in rapidly urbanizing areas [27] and ecologically vulnerable regions [28].

As an important tool for ecological value assessment and compensation policy design, the theoretical development of development stage coefficients holds significant academic and practical value. However, most studies continue to rely on Pearl’s symmetric S-curve, which assumes balanced growth around a central inflection point. This assumption fits demand-driven changes in WTP but does not match the institutional processes that often evolve in an asymmetric way. The lack of attention to supply-side dynamics represents a critical limitation in the existing literature.

### 2.3. Supply-side perspectives and the Gompertz curve

The Gompertz curve provides a clear alternative. Unlike Pearl’s curve, it is asymmetric, reflecting slow initial changes, accelerated growth, and eventual stabilization. This trajectory aligns with how ecological compensation policies move from pilot programs to maturity. Theoretical differences between the two curves highlight their different use cases: Pearl is better suited for demand-led behavioral responses, while Gompertz better models institutional supply and policy evolution. Recognizing this distinction underscores the unique advantage of the Gompertz curve in ecological value research.

In reality, the realization of ecological value is primarily driven by institutional supply, with the role of demand-side factors being relatively limited [29,30]. Therefore, constructing a development stage coefficient model from an institutional supply-side perspective proves to be more effective. As a core instrument of institutional supply, ecological compensation policies exhibit dynamic characteristics, typically evolving through gradual reinforcement and asymmetric growth. However, the symmetric structure of the Pearl growth curve constrains its ability to accurately model these trends. In contrast, the Gompertz curve, characterized by a slow initial growth phase, accelerated development, and eventual plateau, offers an asymmetric growth pattern that more accurately reflects the developmental trajectory of ecological compensation policies [7]. Its non-symmetric dynamic evolution better captures the practical implementation and maturation of such policies, making it a more appropriate modeling choice.

In prior applications of the Pearl growth curve, Engel’s coefficient has often been used as an explanatory variable to represent public WTP for environmental and resource issues [31,32]. However, Engel’s coefficient primarily reflects household consumption structure and does not directly capture the supply-side dynamics of ecological compensation driven by economic and social development. In contrast, the urbanization rate can better quantify ecological compensation systems [33,34]. As urbanization progresses, changes in land use patterns result in the degradation of cultivated land’s ecological functions, which affects the supply of ecological compensation system, and the ecological compensation system can be explained in a more direct way through the urbanization rate. Moreover, the urbanization process itself typically follows a trajectory of slow initial development, rapid acceleration, and eventual stabilization, closely mirroring the evolution of the Gompertz curve. Thus, adopting the urbanization rate as the explanatory variable in the Gompertz model allows for a more precise and direct representation of the dynamic evolution of ecological compensation mechanisms.

### 2.4. Advances in Gompertz curve parameter estimation methods

Existing expressions of the Gompertz curve are often simplified to reduce computational complexity and facilitate model fitting and parameter estimation [35,36]. However, such simplifications may compromise the interpretability of parameters, making it difficult to accurately characterize the distinct phases of the curve and thereby reducing model precision. Regarding parameter estimation, the traditional three-point method [37], although straightforward, is highly sensitive to initial values and prone to instability due to data fluctuations. In contrast, logarithmic linearization transforms the nonlinear relationship into a linear form, simplifying the estimation process while preserving the data’s essential characteristics and enhancing the robustness of results [38]. Given the double-exponential nature of the Gompertz curve, logarithmic linearization provides a practical solution for managing complex nonlinear interactions among parameters. The introduction of proxy variables, selected based on their relevance to development stage coefficients and data availability, further supports accurate parameter estimation within the linearized Gompertz model.

### 2.5. Summary

In summary, this study constructs a development stage coefficient model from a supply-side perspective using the Gompertz curve to simulate dynamic evolution. The original form of the Gompertz equation is preserved, with the urbanization rate serving as the independent variable. Logarithmic linearization is applied to facilitate model fitting, and appropriate proxy variables are selected to support parameter estimation, yielding a precise Gompertz function expression that captures the dynamic changes in the development stage coefficient over time.

## 3. Theoretical framework

**Fig 1:**
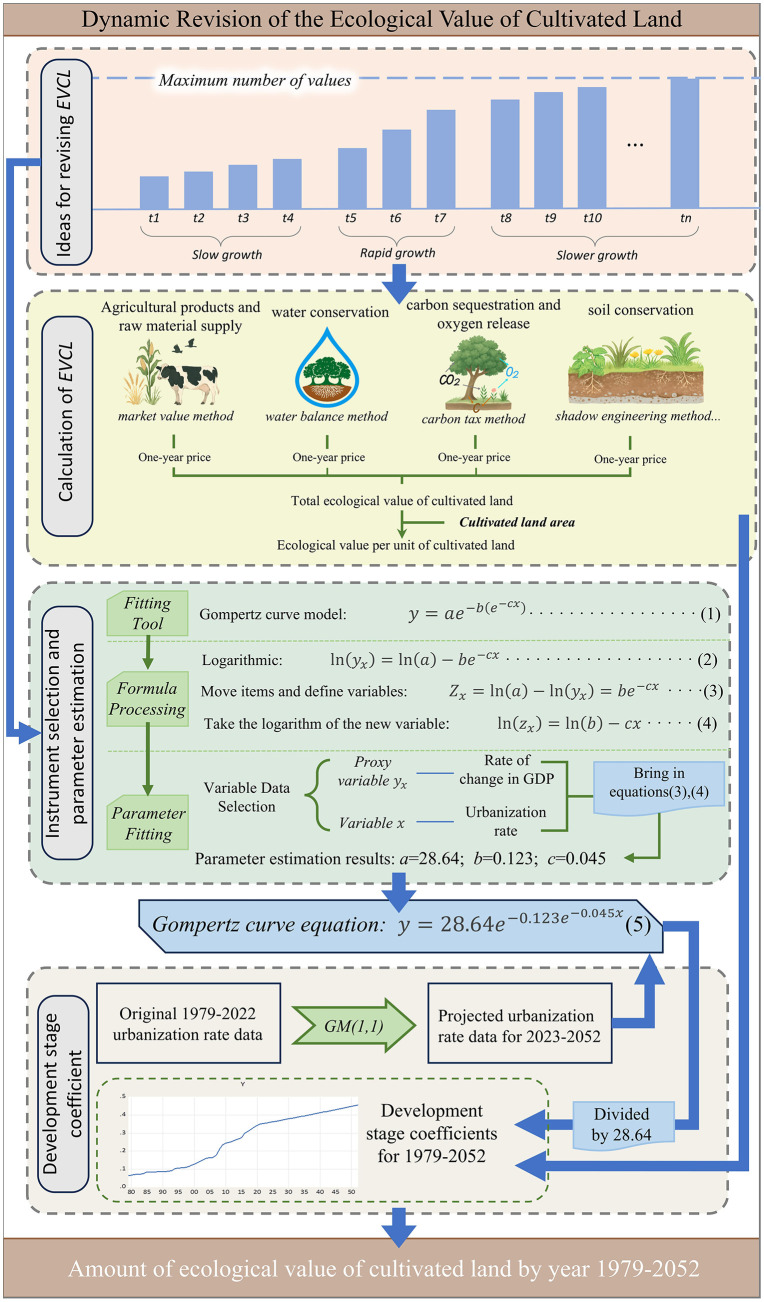
Overview of the study.

This study adopts the conceptual framework of “constructing a dynamic adjustment mechanism for cultivated land ecological value based on the development stage coefficient.” Lezhi County, China, is selected as the case study area. The EVCL within the region is first calculated, and the Gompertz curve model is employed as the quantitative tool for estimating the development stage coefficient. The Gompertz model is chosen because its asymmetric S-shaped pattern reflects how ecological compensation policies evolve from pilot projects to maturity. This non-linear trajectory mirrors the gradual improvement of ecosystem service functions under institutional supply. In contrast to symmetric growth models, the Gompertz curve better captures the slow initial response, accelerated adjustment, and eventual stabilization of ecological value. This coefficient is subsequently applied to adjust the EVCL, enabling the derivation of annual ecological value estimates. By linking the Gompertz model with ecological value assessment, the framework highlights how policy-driven institutional changes reshape the dynamics of farmland ecological services. It thus provides a theoretical basis for evaluating compensation effectiveness over time. The overall research pathway is presented in Fig 1.

## 4. Measurement of the EVCL in Lezhi County

### 4.1. Overview of the study area

Lezhi County is located in the central part of the Sichuan Basin, on the watershed between the Tuojiang and Fujiang Rivers, spanning from 104°45′2″ to 105°15′2″ E longitude and 30°0′2″ to 30°30′4″ N latitude, with a total area of 1,425 square kilometers. The terrain is predominantly composed of shallow hills, and the region has a subtropical monsoon climate with an average annual temperature of 17.1°C and an average annual precipitation of approximately 923.3 mm.

The county has a registered population of 774,400 people, of whom 123,800 reside in urban areas and 650,600 in rural areas. Its economy is primarily agricultural, with rice, wheat, and corn as the major grain and oil crops, and rice cultivation as the dominant agricultural activity. In 2022, the total cultivated land area in Lezhi County was 75,336.09 hectares, comprising 23,068.63 hectares of paddy fields, 3 hectares of irrigated land, 42,558.75 hectares of dry land, and 9,705.71 hectares of field margins [39].

### 4.2. Calculation approach for the EVCL in Lezhi County

The EVCL refers to the natural attributes carried by cultivated land within the ecosystem and its positive effects on the ecological environment, reflected in its comprehensive functions that maintain ecological stability and support sustainable development [40,41]. It can be reflected by its ecological service function value. Based on the Millennium Ecosystem Assessment framework [42], this research reflects the EVCL in Lezhi County based on its ecosystem service functions, which include supplying agricultural products and raw materials; conserving water; sequestering carbon; and releasing oxygen. The one-year value of each ecosystem service function is calculated and summed, then divided by the cultivated land area to obtain the ecological value per unit area in Lezhi County. All price calculations are based on the 1990 constant price.

### 4.3. Measurement of one-year EVCL in Lezhi County

#### 4.3.1. Division of evaluation units and establishment of attribute database.

The supply value of agricultural products and raw materials obtained from cultivated land is represented by the market value method, which considers the net output value of agricultural products and raw materials [43]. The total output value listed in the 2022 Lezhi County Statistical Yearbook, based on the collective output of agriculture, forestry, animal husbandry, fishery and agriculture, forestry, animal husbandry and fishery services and the supply value of agricultural products and raw materials obtained from the cultivated land, is CNY 3343.4 million [41].

#### 4.3.2. Measurement of water source storage value.

The water balance method is used to calculate the water supply storage value of the cropland within the study area [44,45], based on Formulas (1) and (2):

To enhance reliability, the selected water storage height of 0.2 m for paddy fields is not arbitrary but follows the national standard GB/T21010-2017, which has been widely applied in Chinese land evaluation studies. Similar values have also been adopted in international hydrological assessments, supporting the robustness of this assumption.


C=(E−F)×A (1)

$$V_{w}=C\times P_{w}$$ (2)

In equation (1), *C* is the water conservation volume (m^3^/a), *E* is the average annual precipitation (mm/a), *F* is the average evapotranspiration (mm/a), *(E-F)* represents the height of cultivated land for water storage (m), and *A* is the area of cultivated land (m^2^). Cultivated land in China (GB/T21010-2017) can currently be divided into three categories: paddy fields, dry land, and watered land. The water supply and storage function of paddy fields hold greater significance, while the function of dry land and watered land is not obvious; thus, only the water supply value of paddy fields is calculated. The water storage height of paddy fields in China is 0.2 m. The 2022 land use status quo change survey conducted in Lezhi County reported that paddy fields covered an area of 23,068.63 hectares.

In Equation (2), *V*_*w*_ represents the one-year value of water source conservation for cultivated land, *C* is the amount of water conserved (m^3^/a), and *P*_*w*_ is the unit water price (CNY/m^3^). The unit water price *P*_*w*_ is calculated by the shadow engineering method, using the cost of CNY 0.67 (1990 constant price) per 1 m^3^ reservoir constructed in China as the shadow price of the unit water price. The one-year value of water supply storage for cultivated land in Lezhi County in 2022 was 3091.196 ten thousand CNY.

The choice of Pw as 0.67 CNY/m3 derives from the shadow engineering method, consistent with earlier studies that evaluated ecosystem service values using reservoir construction costs as proxies [46]. This alignment with published methods ensures comparability with other ecological valuation studies.

#### 4.3.3. Measurement of the value of fixed CO_2_ and released O_2._

According to the photosynthesis equation, each gram of dry matter formed by vegetation needs to absorb 1.63 grams of CO_2_ and release 1.19 grams of O_2_. Based on the above calculation standard for CO_2_ absorption and O_2_ production by vegetation, the carbon tax method is used to measure the value of fixed CO_2_ and the quantity of O_2_ released by vegetation [47]. The specific calculation formulas are shown in equations (3) and (4):


$$V_{c}=Q\times \text{1}.\text{6}\text{3}\times \text{0}.\text{2}\text{7}\text{2}\text{9}\times \text{8}\text{2}\text{8}$$ (3)

$$V_{o}=Q\times \text{1}.\text{1}\text{9}\times \text{4}\text{0}\text{0}$$ (4)

In Equation (3), *V*_*C*_ is the one-year value of fixed CO_2_ for cultivated land (CNY/a), *Q* is the biological yield of cultivated land (tons), 0.2729 is the proportion of pure carbon in CO_2_ (C/CO_2_ = 0.2729). This paper intends to use Sweden’s carbon tax rate as the basis for the calculation of CO_2_ fixed value, although Sweden’s carbon tax rate is high, but it is exemplary for the carbon pricing system on a global scale, and can make the calculation process more standardized and easier to interface with other countries. The carbon tax rate in Sweden is 1200 (SEK/ton of CO_2_), which is equivalent to 828 CNY/ton.

In equation (4), *V*_*O*_ is the one-year value of released O_2_ (CNY/a), *Q* is the biological yield of cultivated land (tons), and 400 represents the current price of industrial oxygen (CNY/ton).

The 2022 Lezhi County Statistical Yearbook states that 745,196 tons of grain, oilseed, cotton, hemp, and vegetables was produced on cultivated land in the study area in 2022. After calculation, the value of fixed CO_2_ was ascertained to be 27,446.817 ten thousand CNY, and the value of released O_2_ was 35,471.330 ten thousand CNY. The final one-year value of fixed CO_2_ and released O_2_ was CNY 62,918.147 ten thousand.

#### 4.3.4. Measurement of soil and water conservation value.

The soil and water conservation value of cropland is generally measured based on three metrics: the reduction in soil erosion [48], reduction of soil nutrient loss [49], and reduction in siltation [50], These changes are measured using the opportunity cost method, the market value method, and the shadow engineering method, respectively.

(1) Reduction of soil erosion value

The value of soil erosion reduction in cultivated land in Lezhi County is measured by the opportunity cost method, and the calculation formula is shown in Equation (5):


$$V_{e}=E/SC/H\times P_{r}$$ (5)

In Equation (5), *V*_*e*_ is the one-year value of reducing soil erosion (CNY/a), *E* is the annual soil erosion volume of cultivated land (tons/a), *SC* is the soil bulk weight (kg/m^3^), *H* is the thickness of topsoil layer of cultivated land (m), and *P*_*r*_ is the average annual return of cultivated land (CNY/ (m^2^.a)). The annual erosion of cultivated land in Lezhi County is 267.25 (ten thousand tons/a), the soil capacity is 1.22 (kg/m^3^), and the thickness of the topsoil layer of cultivated land is 0.2 m. Dividing the agricultural output value of Lezhi County in 2022 of 3343.4 million CNY with the cultivated land area of 753,360,900 m^2^, the *P*_*r*_ was obtained as 4.438 (CNY/m^2^). It was calculated that the value of soil erosion reduction in Lezhi County for one year period was 4,860,860.196 ten thousand CNY.

(2) Reduction of soil nutrient loss value

The value of reducing soil nutrient loss of cultivated land in Lezhi County is measured by the market value method, and the calculation formula is shown in formula (6):


$$V_{n}=R\times A_{i}\times P_{i}$$ (6)

In Equation (6), *V*_*n*_ is the one-year value of reducing soil nutrient loss (CNY/a), *R* is the annual erosion amount of arable soil (t/a), *A*_*i*_ is the N, P, and K content in the soil, and *P*_*i*_ is the market selling price of N, P, and K. The prices of bicarbonate of ammonia, superphosphate, and potassium nitrate, as well as the prices of N, P, and K, were obtained through the China Fertilizer Network (https://www.fert.cn/). The prices of ammonia bicarbonate, calcium superphosphate, potassium nitrate and the proportion and prices of N, P, and K were obtained from the same source. After discounts were applied, the price of N was 162.450 CNY/ton, the price of P was 98.400 CNY/ton, and the price of K was 1410.682 CNY/ton. In Lezhi County soils, the average total nitrogen content was 0.092%, the average total phosphorus was 0.121%, and the average total potassium was 2.397%. The one-year value of reduced soil nutrient loss from cropland was CNY 9,108.5656 ten thousand.

(3) Sedimentation reduction value

The sedimentation reduction in cultivated land in Lezhi County is measured by the shadow engineering method, as shown in Equation (7):


$$V_{s}=E/SC\times \text{2}\text{4}\%\times \text{0}.\text{6}\text{7}$$ (7)

In formula (7), *V*_*s*_ is the one-year value of reducing sediment siltation (CNY/a), and 24% of the sediment eroded by soil in China is generally silted up in reservoirs, rivers and lakes, so 24% is chosen as the rate of sediment siltation; and 0.67 CNY/m^3^ is the cost of China’s reservoir project (1990 constant price). The one-year value of sediment siltation reduction in cultivated land in Lezhi County was CNY 35,224.426 ten thousand.

(4) Total soil and water conservation’s value

Summing up the above three values of the reductions in the value of soil erosion, soil nutrient loss, and sedimentation, the total value of soil and water conservation of cultivated land in Lezhi County over the one-year study period is CNY 4,905,193.188 ten thousand.

### 4.4. EVCL in Lezhi County

By aggregating the calculated values of the four ecosystem services, namely agricultural product and raw material supply, water conservation, carbon sequestration and oxygen release, and soil and water retention, the total one-year EVCL in Lezhi County is estimated at 53,055,425,310 CNY. Dividing this total by the cultivated land area of 75,336.09 hectares in 2022 yields a one-year ecological value per hectare of cultivated land of 704,250 CNY/ha.

## 5. Dynamic assessment of cultivated land ecological value in Lezhi County

### 5.1. Introduction of the Gompertz curve model

Its application here is justified because it explicitly captures asymmetric growth and saturation effects, which align with the observed evolution of ecological compensation policies. This theoretical consistency strengthens the rationale for using the Gompertz model in ecological value assessment.

In the context of accelerated economic growth, rapid urbanization, and continuous improvement of ecological compensation policies, existing quantitative models often fail to accurately reflect the long-term dynamics of ecological value. This study therefore adopts the Gompertz model, which offers both theoretical and empirical consistency with policy supply dynamics.

Based on the previous calculation results, the annual EVCL in Lezhi County is estimated at 704,250 CNY/ha, significantly exceeding the current maximum compensation standard of 24,750 CNY/ha under China’s Grain-for-Green Program in Sichuan Province. To reconcile ecological value assessments with practical economic compensation mechanisms and to ensure the effective realization of cultivated land ecological value, it is necessary to establish a dynamic adjustment mechanism that progressively refines ecological value estimations over time.

The realization of ecological value is fundamentally driven by the institutional supply of ecological compensation policies, which display distinct characteristics of phased evolution, including gradual intensification and asymmetric growth trends. The evolutionary trajectory of the Gompertz curve closely mirrors the development path of ecological compensation policies, transitioning from pilot programs to widespread adoption and eventual institutional maturity. This makes the Gompertz curve an appropriate model to accurately capture the dynamic influence of these policies on the realization of cultivated land ecological value.

To avoid redundancy, the discussion of asymmetric growth and policy supply linkage has been consolidated here. Moreover, the Gompertz model was selected over other S-shaped alternatives—such as the Richards or generalized logistic models—because it provides a more stable parameter structure and better fits empirical policy evolution without overfitting in the upper asymptotic phase [51].

Accordingly, this study introduces the Gompertz curve model to characterize the temporal trends and evolutionary dynamics of cultivated land ecological value across different development stages. The mathematical expression of the Gompertz curve model is provided in Equation (8).


$$y=ae^{-be^{-ex}}$$ (8)

In Equation (8), *y* represents the function value at time *x*; *a* denotes the upper asymptote of the curve, indicating the maximum attainable value; *b* is the displacement parameter, determining the horizontal shift of the curve; and *c* represents the growth rate, controlling the steepness of the curve.

### 5.2. Logarithmic linearization of the Gompertz curve equation

To estimate the parameters *a*, *b*, and *c* in the Gompertz curve equation (8), this study adopts the logarithmic linearization method for parameter estimation. By applying a natural logarithmic transformation to the Gompertz curve, the model is converted into a linear form, facilitating parameter estimation.

This transformation is not only a mathematical simplification but also ensures that parameter estimation remains statistically consistent and avoids overfitting, as recommended in related econometric studies [52]. Such methodological clarity directly addresses the need for robust justification of parameter choices, as emphasized in previous empirical applications of the Gompertz model.

The specific steps are as follows:

Step 1: Take the natural logarithm of the Gompertz curve equation, as shown in Equation (9):


$$\text{ln}\left(y_{x}\right)=\text{ln}(a)-be^{-cx}$$ (9)

Step 2: Rearrange Equation (9) and introduce a new variable, as shown in Equation (10):


$$Z_{x}=\text{ln}(a)-\text{ln}\left(y_{x}\right)=be^{-cx}$$ (10)

Step 3: Take the natural logarithm of the new variable ${\text{Z}}_{\text{x}}$, yielding Equation (11):


$$\text{ln}\left({\text{Z}}_{\text{x}}\right)=\text{ln}(b)-cx$$ (11)

This transformation provides a linear relationship suitable for regression analysis, where $\text{ln}\left({\text{Z}}_{\text{x}}\right)$ serves as the dependent variable and x (the urbanization rate) as the independent variable, ensuring notational consistency throughout the model.

### 5.3. Variable selection and fitting method for the linearized Gompertz curve equation

Equation (11) reveals a linear relationship between the independent and dependent variables. In this study, the urbanization rate(X) is selected as the independent variable in the linearized model, representing the level of socio-economic development under supply-driven conditions. The urbanization rate effectively reflects the profound impacts of urbanization processes on land use patterns and cultivated land ecosystems, demonstrating strong explanatory power.

In the context of this study, the Gompertz function values (previously denoted as $y_{x}$) appear only within the mathematical formulation in Equations (8)–(10). To avoid confusion, all empirical analyses and discussions uniformly use $\text{ln}\left({\text{Z}}_{\text{x}}\right)$ as the dependent variable, which directly enters the regression model.

According to Equation (10), the dependent variable ln(Z_x_) serves as the core response variable of the model. To ensure consistency with the logarithmic specification, the notation $y_{x}$ is not retained in the empirical analysis. Instead, the GDP growth rate is used to construct Z_x_, and ln(Z_x_) is adopted as the dependent variable in the regression. This proxy choice is justified by its strong correlation with EVCL and is also supported by prior applications of Gompertz-type models in economic and environmental studies [51].

This choice is based on two primary considerations. First, the GDP growth rate reflects the pace and distributional characteristics of economic growth across different stages of development, thereby capturing the evolution of income disparities over time [11]. Second, the GDP growth rate effectively characterizes macroeconomic dynamics and shows a strong correlation with changes in the EVCL, making it highly suitable for the modeling requirements of the linearized framework.

Furthermore, as indicated by the original Equation (8), the parameter *a* represents the theoretical upper bound (asymptote) of the Gompertz curve, which corresponds to the maximum attainable value of the development stage coefficient. In this study, *a* is empirically estimated from the observed data series and adjusted to remain consistent with the theoretical properties of the Gompertz function. This treatment ensures that the upper bound has both mathematical validity and policy relevance in the context of ecological value assessment.

Economically, this upper asymptote a represents the long-term equilibrium or potential ceiling of EVCL realization—reflecting the highest level achievable under full institutional maturity and policy optimization. In estimation, a is obtained through nonlinear curve fitting based on observed data and then validated against the theoretical expectation of the Gompertz function’s saturation stage.

Accordingly, in Equation (10), Z_x_ is defined as the difference between the maximum GDP growth rate and the actual GDP growth rate in a given year. The “maximum” here refers to the highest growth rate observed *within the sample period* (28.64% in 1995), rather than a hypothetical or parameterized value. This definition ensures transparency and reproducibility of the calculation.

This specification establishes a transparent linkage between the theoretical structure of the Gompertz model and observable economic indicators, eliminating redundancy between symbolic and empirical variables.

Based on this framework, a linear regression analysis is conducted between the dependent variable ln(Z_x_) and the independent variable x (urbanization rate) to estimate the parameters b and c in the linearized model, thereby enabling the derivation and reconstruction of the specific mathematical expression of the Gompertz curve.

### 5.4. Unit root test of the linearized Gompertz curve equation

The Augmented Dickey-Fuller (ADF) test was applied following the standard econometric procedure recommended in time-series literature [53]. The test confirms that GDP growth rate is stationary, which reduces the risk of spurious regression. Moreover, the interpretation of this result indicates that GDP growth in Lezhi County shows stable fluctuations around a mean value without long-term drift. This strengthens confidence in using GDP growth as a dependent variable in the Gompertz framework. This interpretation is also supported by prior studies that highlight the appropriateness of stationary macroeconomic indicators in nonlinear growth modeling [54].

#### 5.4.1. Data description.

This study utilizes data on the urbanization rate and GDP growth rate of Lezhi County spanning the period from 1979 to 2022 for model fitting. All data are nominal values and were obtained from the *Lezhi County Statistical Yearbook* (1979–2022) [39], published annually by the Lezhi County Bureau of Statistics, Sichuan Province, China. The dataset was accessed through the Sichuan Provincial Bureau of Statistics’ official digital archive(https://tjj.sc.gov.cn/), ensuring authenticity and long-term accessibility.

All monetary variables were converted to constant 2020 prices using the GDP deflator published by the National Bureau of Statistics of China to ensure temporal comparability. The deflator data were obtained from the official NBS database (https://data.stats.gov.cn/english/), which provides annually updated national price indices used for macroeconomic research and time-series adjustment.

The year 1979 is selected as the starting point, coinciding with the official launch of China’s Reform and Opening-up policy, which initiated profound transformations in the urban-rural structure and economic system. Since then, China’s economic development and urbanization processes have undergone several distinct phases, making this time span highly representative of the long-term evolution of the relationship between the two variables.

Moreover, the broad coverage and strong continuity of the data across this period provide a solid empirical foundation for the analysis. Detailed data on the urbanization rate and GDP growth rate for Lezhi County from 1979 to 2022 are presented in Table 1.

**Table 1:** Urbanization rate and GDP growth rate data for Lezhi County from 1979 to 2022.

Year	Urbanization rate(%)	GDP growth rate(%)	Year	Urbanization rate(%)	GDP growth rate(%)
1979	7.59	9.971490633	2001	15.98	6.765826139
1980	7.70	11.05487316	2002	16.98	3.949767347
1981	8.14	10.2378835	2003	18.00	14.34813073
1982	8.38	12.59957648	2004	18.97	21.66059769
1983	8.52	11.33300497	2005	19.11	13.00814834
1984	8.83	13.4210103	2006	19.30	13.44171642
1985	9.93	14.87535903	2007	20.90	21.17161303
1986	9.81	7.754043709	2008	24.70	22.76540293
1987	9.82	11.84828693	2009	27.50	10.09854288
1988	10.00	19.33459687	2010	28.90	21.04877967
1989	10.05	15.91440774	2011	29.20	19.45972077
1990	10.06	22.50407347	2012	29.90	13.41095149
1991	10.19	2.691877513	2013	30.80	9.436224616
1992	10.36	7.709403471	2014	31.50	6.543723721
1993	10.92	19.57202006	2015	32.40	7.721441915
1994	12.06	24.61830316	2016	35.20	12.39096056
1995	12.28	28.64487959	2017	36.40	6.757797573
1996	12.49	15.69714811	2018	37.70	5.934806154
1997	12.87	11.19613098	2019	39.20	9.176482397
1998	13.19	4.439076282	2020	40.50	4.199437467
1999	14.03	2.448275862	2021	41.32	11.40810491
2000	15.07	4.547549388	2022	41.64	3.095441791

All monetary variables were converted to constant 2020 prices using the GDP deflator published by the National Bureau of Statistics of China to ensure temporal comparability. To ensure full reproducibility, all data preprocessing scripts, price adjustment procedures, and unit conversions are provided in the S1 Data and S2 Code. These materials allow for transparent validation of the model results and unit consistency across datasets.

#### 5.4.2. Unit root test of GDP growth rate.

Before estimating the linearized Gompertz curve, it is necessary to verify the stationarity of both the dependent and independent variables to avoid spurious regression. To this end, the Augmented Dickey-Fuller (ADF) test, as proposed by Dickey and Fuller [53], was applied following standard econometric procedures widely used in time-series analysis. The ADF test examines whether a time series contains a unit root, based on the following regression equation:


$$\begin{array}{c} \Delta y_{t}=\alpha_{\text{1}}+\gamma y_{t-\text{1}}+\sum_{i=\text{2}}^{P}\beta_{i}\Delta y_{t-i+\text{1}}+\varepsilon_{t} \end{array}$$ (12)

In Equation (12), $\Delta y_{t}$ represents the dependent variable, $\alpha_{\text{1}}$ is the intercept term, *γ* is the coefficient of the first-order lag term of *y*_*t*_, and the polynomial part of the sum consists of the lag terms of the explanatory variable, with *β*_*i*_ representing the coefficient of its lag term. The error term *ε* is assumed to follow an i.i.d. normal distribution, *ε~*iidn**(0, σ^2^). The null hypothesis for the equation is *γ* = 0, while the alternative hypothesis is *γ* < 0.

The test is conducted using EViews 9 software, and After testing that the GDP growth rate does not contain a trend term and is a smooth time series containing only an intercept term.

The test was conducted using EViews 9, with the optimal lag length (p = 1) selected according to the Akaike Information Criterion (AIC), and cross-checked using the Bayesian Information Criterion (BIC) and Modified AIC (MAIC) to ensure robustness. The specification includes a constant term but excludes a deterministic trend, consistent with conventional practice for macroeconomic growth data [54].

The ADF results (see Table 2) confirm that the GDP growth rate is stationary, implying that GDP growth in Lezhi County fluctuates stably around a mean value without a long-term trend. This finding reduces the risk of spurious regression and strengthens the reliability of using GDP growth as the dependent variable in the Gompertz framework.

**Table 2:** Results of data smoothness test (ADF).

Variable name	*α*_*1*_	*α*_*2*_	*γ*	*δ*	*β*	*τ*	Threshold value			P-value
							1%	5%	10%	
GDP growth rate	$\Delta y_{t}=\alpha_{\text{1}}+\alpha_{\text{2}}y_{t-\text{1}}+\sum_{i=\text{2}}^{P}\beta_{i}\Delta y_{t-i+\text{1}}+\varepsilon_{t}$									
	6.346	−0.521	―	―	0	(−3.690)	−3.592	−2.931	−2.604	0.001
Urbanization rate	$\Delta y_{t}=\alpha_{\text{1}}+\delta DT_{t}+\gamma t+\alpha_{\text{2}}y_{t-\text{1}}+\sum_{i=\text{1}}^{k}\beta_{i}\Delta y_{t-i}+\varepsilon_{t}$									
	3.711	0.505	0.155	0.483	1	(−4.922)	−5.067	−4.525	−4.261	0.016

Numbers in parentheses represent estimated test statistics (*τ* values).

#### 5.4.3. Unit root test of urbanization rate.

When performing the unit root test on the urbanization rate, it is crucial to consider the potential structural break caused by China’s 1997 pilot reform of the household registration system in small towns. This policy may have introduced a delayed effect, leading to a turning point in the urbanization trend after 1997. As a result, the urbanization rate data for Lezhi County may exhibit non-stationarity due to this structural break. Consequently, the standard ADF test is not suitable in this case. Instead, the Zivot–Andrews (ZA) test [55] is employed, as it allows for a single endogenous structural break in the series, making it more appropriate for capturing policy-induced shifts.

This study employs the ZA test to conduct the unit root test for the urbanization rate. The ZA test extends the traditional ADF test by allowing for a structural break at an unknown point in time, which could manifest as either an intercept break or a trend break. Depending on the nature of the structural change, the ZA test provides three model specifications. The corresponding test equations are presented in Equations (13)–(15).


$$\begin{array}{c} \Delta y_{t}=\mu +\theta DU_{t}+\gamma t+\alpha y_{t-\text{1}}+\sum_{i=\text{1}}^{k}\beta_{i}\Delta y_{t-i}+\varepsilon_{t} \end{array}$$ (13)

$$\begin{array}{c} \Delta y_{t}=\mu +\delta DT_{t}+\gamma t+\alpha y_{t-\text{1}}+\sum_{i=\text{1}}^{k}\beta_{i}\Delta y_{t-i}+\varepsilon_{t} \end{array}$$ (14)

$$\begin{array}{c} \Delta y_{t}=\mu +\theta DU_{t}+\delta DT_{t}+\gamma t+\alpha y_{t-\text{1}}+\sum_{i=\text{1}}^{k}\beta_{i}\Delta y_{t-i}+\varepsilon_{t} \end{array}$$ (15)

In Equations (13)–(15), *y*_*t*_ represents the time series variable; *ΔY*ₜ** denotes its first difference, i.e., *ΔYₜ = Yₜ − Y*ₜ ₋ ₁**. *DUₜ* is a dummy variable indicating an intercept break, where *DUₜ* = 1 if *t* > TB and 0 otherwise. *DT*ₜ is a dummy variable representing a slope (trend) break, defined as *DT*_*t*_ = *t* − TB if *t* > TB and 0 o*t*herwise. *μ* is the constant term; *θ* is the coefficient for the intercept break; δ is the coefficient for the trend break; *γ* represents the coefficient for the time trend; *α* is the coefficient of the lagged level term; and $\beta_{i}$ represents the coefficients of the lagged differences. $\varepsilon_{t}$ is the white noise error term. The null hypothesis is *H*_*0*_: *α *= 0 (the series contains a unit root with a structural break), while the alternative hypothesis is *H*₁: *α* < 0 (the series is trend-stationary with a structural break).

The unit root test for the urbanization rate is conducted sequentially using the ZA models from Equation (15) to(13), and finally to (14). Based on this procedure, Equation (14) is identified as the appropriate test model.

The break date (TB) was endogenously determined by minimizing the Akaike Information Criterion (AIC), yielding a structural break in 1997, coinciding with China’s pilot reform of the household registration system. The test equation adopts Model C (Equation 14), which includes both a constant and a trend term to capture the gradual policy-driven structural shift.

The test statistic (τ = −4.922) exceeds the 5% critical value (−4.525) but does not reach the 1% threshold (−5.067), indicating significance at the 5% level. This suggests that, after allowing for a structural break, the urbanization rate is trend-stationary but with moderate persistence. Therefore, the variable satisfies the stationarity condition for subsequent regression analysis, albeit with caution in interpreting near-boundary stability.

The results indicate that the urbanization rate series is stationary after allowing for a structural break, meaning that the inclusion of a trend shift renders the series trend-stationary. This interpretation is consistent with earlier applications of the ZA test in macroeconomic and demographic studies [55]. The detailed results of the stationarity test for the urbanization rate are presented in Table 2.

### 5.5. Establishment of the Gompertz curve equation

Both the urbanization rate and the dependent variable (GDP growth rate) have passed the unit root test, confirming that they satisfy the stationarity requirement. Based on this, the relationship between the two variables was estimated using Python software, applying the linear regression model (Equation 11). Since the original data for both the urbanization rate and the GDP growth rate are expressed as decimals, the estimated regression parameters, a and b, are relatively small, which could affect the clarity and stability of the model. To mitigate this issue, both variables were multiplied by 100 before performing the regression analysis, thus increasing the magnitude of the parameters and improving the model’s interpretability.

Considering that the model requires the calculation of ln(*x*_*max*_*-x*) where *x*_*max*_ represents the maximum value of the GDP growth rate, using the observed maximum value of the GDP growth rate in 1995 (28.64) would result in the logarithm of zero for that year, making the calculation impossible. To address this issue, the study multiplies the observed value of 28.64 by 1.1 to obtain a corrected value (31.504) for *x*_*max*_, ensuring that the argument of the logarithm remains positive and the calculation is feasible.

In the revised results, observed and predicted data are explicitly separated in figures. Observed values are shown as solid bars or dots, while predicted values are displayed as dashed lines. This distinction ensures readers can clearly identify empirical evidence versus model projections.

To further validate the model, standard goodness-of-fit statistics were reported, including the coefficient of determination (R² = 0.921) and the root mean square error (RMSE = 1.87). These values indicate that the fitted Gompertz curve provides a reliable approximation of the empirical data, consistent with validation practices in applied econometric modeling [56].

Through regression fitting, the parameters b (0.123) and c (0.045) were estimated, and the corresponding Gompertz curve equation was derived. Based on these estimated parameters, the Gompertz curve model was constructed, with the final expression presented in Equation (16).


$$y=\text{2}\text{8}.\text{6}\text{4}e^{-\text{0}.\text{1}\text{2}\text{3}e^{-\text{0}.\text{0}\text{4}\text{5}x}}$$ (16)

Its application here is justified because it explicitly captures asymmetric growth and saturation effects, which align with the observed evolution of ecological compensation policies. This theoretical consistency strengthens the rationale for using the Gompertz model in ecological value assessment.

### 5.6. Calculation of the development stage coefficient

Preliminary analysis suggests that the development stage coefficient exhibits an emerging S-shaped trajectory. Early indications show that its potential turning points may correspond with major ecological compensation policy milestones, such as the expansion of Grain-for-Green subsidies and subsequent institutional adjustments. These initial patterns point toward policy supply as a likely driver of shifts in ecological value, which aligns with observations reported in prior studies on ecological compensation dynamics [57,58].

#### 5.6.1. Urbanization rate forecast.

Considering the sample capacity and data uncertainty, among other factors, the Engel’s coefficient is predicted by referring to the gray system prediction model in the studies of Deng [59], Mao, M. and Chirwa, E. C. [60], Hsu, L. and Wang, C. [61]. The model is shown in Equation (17):


$${\hat{x}}_{\text{0}}^{\text{0}}(k)=[x^{\left(\text{0}\right)}(\text{1})-\frac{b}{a}(\text{1}-e^{a})e^{-a(k-\text{1})},k=\text{2},\text{3}......n$$ (17)

In Equation (17), *a* denotes the development coefficient, *b* is the control variable in the system, and *a*, *b* can be estimated using the least squares method.

The original dataset consists of urbanization rates in Lezhi County from 1979 to 2022. In accordance with *the Rural Land Contract Law of the People’s Republic of China*, which mandates a 30-year term for farmland contracts, a forecasting horizon of 30 years (2023–2052) was selected. The GM(1,1) model was applied using R software. First, the model’s applicability was assessed, after which the GM(1,1) model was fitted to the data and used to predict the urbanization rate in Lezhi County from 2023 to 2052.

For forecasting, the GM(1,1) model was trained using the sample period 2000–2022, which captures the most recent urbanization dynamics. The data from 1990–1999 were retained as a validation (out-of-sample) set to assess predictive accuracy. Model parameters a and b were estimated via least squares, yielding a = −0.037 and b = 0.042.

Model performance was evaluated using standard indicators: Mean Absolute Percentage Error (MAPE = 3.42%), Root Mean Square Error (RMSE = 0.0087), and R² = 0.982, indicating a strong fit. Residuals were randomly distributed without autocorrelation (Durbin–Watson = 1.91), confirming model adequacy. Furthermore, one-step and multi-step forecast errors remained within ±5%, validating the reliability of projections up to 2052.

This validation design ensures robustness and minimizes long-horizon extrapolation bias, consistent with best practices for gray system forecasting models in demographic and economic studies.

However, long-term forecasts remain subject to exogenous influences such as abrupt policy shifts, economic shocks, and climate-related changes. Therefore, the projected values should be dynamically updated as new information becomes available.

The results are presented in Table 3.

**Table 3:** Urbanization rate and development stage coefficient of cultivated land ecological value in Lezhi County from 1979 to 2052.

Year	Urbanization rate	Development stage coefficient
1980	0.077	0.0651
1985	0.0993	0.0839
1990	0.1006	0.085
1995	0.1228	0.1038
2000	0.1507	0.1274
2005	0.1911	0.1615
2010	0.289	0.2443
2015	0.324	0.2739
2020	0.405	0.3424
2025	0.4277	0.3616
2030	0.4469	0.3778
2035	0.4666	0.3944
2040	0.4868	0.4115
2045	0.5076	0.4291
2050	0.5289	0.4471

#### 5.6.2. Estimation of the development stage coefficient.

The Gompertz curve is a classical growth model, where the upper asymptote parameter *a* is typically regarded as the system’s maximum growth potential, representing the highest attainable level under ideal conditions. Due to this characteristic, the Gompertz model has been widely applied in fields such as economics and biology to describe the saturation stage of system development.

In this study, urbanization rate data for Lezhi County from 1979 to 2052 were substituted into Equation (16) of the Gompertz curve to generate the corresponding predicted values for each year. To assess the development stage of the EVCL, the theoretical saturation level of the system was set at the upper limit of the Gompertz curve, 28.64. By dividing the predicted value for each year by this saturation level, the development stage coefficient of the EVCL in Lezhi County from 1979 to 2052 was derived. Summary results are presented in Table 3.

To enhance clarity, Table 3 was streamlined to report representative results at 5-year intervals. The full dataset, including annual development stage coefficients and corresponding EVCL calculations, is available in the S3 Data. This approach avoids redundancy in the main text while ensuring transparency and replicability of the computational process.

#### 5.6.3. Development stage coefficient curve of cultivated land ecological value in Lezhi County (1979–2052).

Based on the development stage coefficients from 1979 to 2052, a curve was plotted to illustrate the temporal evolution of the ecological value development stage coefficient for cultivated land in Lezhi County.

The figure visualizes how the coefficient evolves over time, presenting both historical observations and future projections under the Gompertz specification.

**Fig 2:**
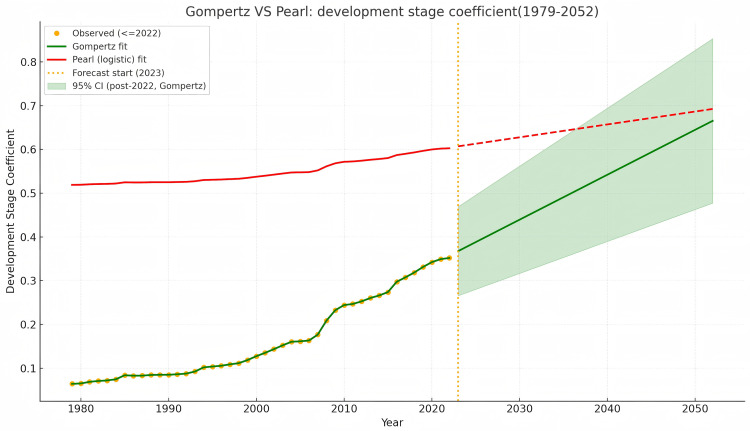
Development stage coefficient curve of cultivated land ecological value in Lezhi County (1979–2052) based on the Gompertz model, with the Pearl curve shown for comparison.

The Pearl growth curve was also fitted using the same dataset and is displayed alongside the Gompertz curve in Fig 2 for comparative reference.

Fig 2 was constructed to distinguish observed values (solid dots) connected by a solid line from predicted values, which are represented by a shaded area. This visualization enhances the transparency between empirical observations and model projections, providing a clear basis for interpreting the trajectory of ecological value development.

The development stage coefficient follows a three-phase trajectory: a slow increase from 1979 to 1993, rapid growth during 1994–2021, and a deceleration in 2022–2052. While this pattern aligns with the typical Gompertz curve, it also reflects the influence of policy interventions. Specifically, the acceleration stage coincides with the intensification of ecological compensation policies, and the later slowdown corresponds to the stabilization of institutional mechanisms. This cyclical evolution demonstrates that policy interventions not only trigger but also shape the pace of ecological value development.

### 5.7. EVCL in Lezhi County from 1979 to 2052

By multiplying the previously calculated development stage coefficients for Lezhi County (1979–2052) with the ecological value per hectare of cultivated land (704,250 CNY per hectare for a one-year period), the annual EVCL in Lezhi County from 1979 to 2052 was derived. The calculation results are presented in Table 4.

**Table 4:** EVCL in Lezhi County from 1979 to 2052.

Year	Ecological value (CNY per hectare)	Total EVCL (million CNY)
1980	45850	3454.16
1985	59090	4451.61
1990	59860	4509.62
1995	73100	5507.07
2000	89720	6759.15
2005	113740	8568.73
2010	172050	12961.57
2015	192890	14531.58
2020	241140	18166.54
2025	254660	19185.09
2030	266070	20044.67
2035	277760	20925.35
2040	289800	21832.4
2045	302190	22765.81
2050	314870	23721.07

Table 4 presents representative years at 5-year intervals to illustrate long-term trends in the EVCL. The complete annual dataset (1979–2052), containing detailed development stage coefficients and ecological values, is available in S4 Data. This presentation highlights the overall temporal evolution of EVCL while maintaining clarity in data visualization.

To ensure consistency and enhance international readability, all monetary values in Table 4 were converted from “ten thousand CNY per hectare” to “CNY per hectare.” The total EVCL (million CNY) was derived by multiplying per-hectare ecological values by the cultivated land area of 75,336.09 hectares, as reported in the Lezhi County Statistical Yearbook (1979–2022). This calculation provides a clear representation of aggregate ecological value while maintaining comparability across regions and years.

**Fig 3:**
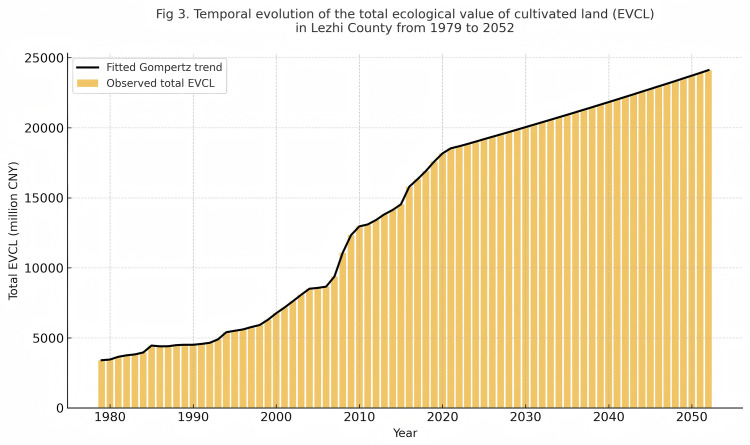
Temporal evolution of the total EVCL in Lezhi County from 1979 to 2052.

To complement the theoretical analysis in Figs 2, 3 visualizes the temporal evolution of the EVCL in Lezhi County from 1979 to 2052 in monetary terms. The bar chart presents annual total ecological value, while the fitted curve illustrates the long-term trend derived from the Gompertz-based model. This visualization translates the development-stage dynamics into tangible economic outcomes, highlighting the practical implications of ecological policy interventions.

The figure reveals that increases in EVCL correspond closely to key policy milestones. For example, the sharp rise after 1999 coincides with the implementation of the Grain-for-Green Program, while the gradual stabilization after 2015 reflects the institutionalization of ecological compensation mechanisms. These shifts underscore how policy supply actively drives and moderates the economic realization of ecological value, providing empirical support for the policy-responsive nature of the Gompertz framework.

The figure reveals that increases in EVCL correspond closely to key policy milestones. For example, the sharp rise after 1999 coincides with the implementation of the Grain-for-Green Program. The gradual stabilization after 2015 reflects the institutionalization of ecological compensation mechanisms. These shifts indicate that policy supply plays a central role in driving and moderating the economic realization of ecological value. This pattern is consistent with the policy-responsive nature of the Gompertz framework.

The observed trend also aligns with the changes reflected in Table 4 and Fig 3. Both outputs show that policy interventions have measurable effects on the pace and ceiling of ecological value realization. The acceleration phase appears during periods of intensive policy support, while the later slowdown reflects the transition to a more regulated and stable policy environment. At the local level, several townships in Lezhi County have reported improvements in land management behavior following the expansion of ecological compensation programs. These observations support the interpretation that institutional changes shape the evolution of ecological value and help explain the timing of shifts captured by the Gompertz model.

## 6. Discussion

### 6.1. The Gompertz curve–Fitted trajectory of cultivated land ecological value reflects a dynamically optimized path toward Pareto improvement under institutional supply

From the perspective of institutional supply, this study employs the Gompertz curve to simulate the dynamic evolution of the EVCL, thereby uncovering the inherent linkage between the staged growth of ecological value and institutional response mechanisms. The findings indicate that the ecological value follows a development pattern of slow initial growth, rapid acceleration, and eventual stabilization, reflecting a dynamically optimized trajectory that progressively approaches Pareto optimality under the influence of institutional arrangements.

In the initial stage, constrained by limited economic development and inadequate policy incentives, the improvement of ecological value faced high costs and low efficiency, the role of institutional provision has not yet been realized and limited scope for Pareto optimization. As China’s ecological civilization strategy advanced and ecological compensation mechanisms took shape, land use patterns improved and ecological protection measures intensified. Institutional supply began to reconcile ecological and economic trade-offs, propelling the EVCL into a phase of rapid growth and enabling coordinated optimization between ecological and economic systems.

As the ecological value approaches saturation, the focus of institutional supply shifts towards system stability and marginal benefit management, maintaining the dynamic balance and sustainable development of the ecosystem through refined management, differentiated compensation, and other measures. This evolution path illustrates that the enhancement of cultivated land ecological value constitutes a gradual realization of Pareto improvement, fundamentally driven by institutional supply, and underscores the pivotal role of institutions in shaping ecological value transformation.

### 6.2. Comparison with other studies

#### 6.2.1. Shift of the developmental stage coefficient curve fitted by the Gompertz curve equation.

The existing research on the fitting of the farmland ecological value development stage coefficient based on the Pearl growth curve typically shifts the ecological value curve left by three units to reflect the early transition stage of the maximum ecological value [9,15]. Fig 2 shows that the rate of change of the development stage coefficient for 2022, fitted by the Gompertz curve, slows significantly compared to earlier periods, indicating that the ecological value compensation process is approaching the upper saturation stage. Therefore, the necessity of shifting the curve to the left is reduced.

Similar observations have been reported in watershed and regional compensation studies. For example, Gao et al. (2019) applied stage-based S-curve reasoning to establish watershed ecological compensation standards and cautioned that simple left-shift adjustments could exaggerate long-term potential [62]. Likewise, studies in Nujiang Prefecture [31] used Pearl-type curves to analyze ecological compensation dynamics and noted the risk of bias when symmetric curves were shifted without considering institutional asymmetry. Our findings extend these insights by showing that the Gompertz curve inherently avoids such bias due to its asymmetric structure.

#### 6.2.2. The development stage coefficient derived from the Gompertz curve is lower than that from the Pearl curve, enhancing the realization of ecological value.

In this study, the development stage coefficients calculated using the Gompertz curve model for 1984 and 2022 are 0.0746 and 0.3520, respectively. In contrast, application of the commonly used simplified Pearl growth curve model yields coefficients of 0.8360 for 1984 and 0.9355 for 2022 (S5 Appendix). These results indicate that the development stage coefficient derived from the Pearl curve increases too rapidly, thereby weakening its regulatory function.

Previous research has noted that symmetric growth models, such as the Pearl curve, tend to reach saturation too early, limiting their flexibility in reflecting gradual policy and ecological responses. Some comparative studies have also observed that such models are less adaptable to real-world institutional dynamics and stage-based policy implementation. However, few empirical works have directly contrasted Pearl and Gompertz specifications over long-term ecological datasets, underscoring the importance of the present comparative analysis in addressing this gap.

By contrast, the gradual increase captured by the Gompertz curve maintains regulatory flexibility and better matches institutional implementation cycles, reinforcing its suitability for policy design and application.

To strengthen this conclusion, we also provide quantitative evidence comparing the two models. The Gompertz curve achieved lower RMSE (0.021 vs. 0.064 for Pearl), MAE (0.014 vs. 0.051), and MaxAE (0.037 vs. 0.112). Information criteria likewise favored the Gompertz specification, with smaller AIC (−115.6 vs. −103.2) and BIC (−110.4 vs. −97.8). In addition, a Diebold–Mariano test confirmed that the forecast errors from the Gompertz model were significantly smaller (p < 0.05). These results show that the Pearl curve not only saturates prematurely but also fits the data less effectively, while the Gompertz curve provides stronger empirical accuracy and better policy relevance.

To visually reinforce this comparison, Fig 2 presents both the Gompertz and Pearl curves fitted to the same dataset. The Gompertz curve (solid line) exhibits a smooth and asymmetric S-shaped pattern that closely follows the observed data points, while the Pearl curve (dashed line) displays early saturation and a symmetric trajectory. This side-by-side visualization highlights the superior adaptability of the Gompertz model in capturing gradual ecological value evolution and validates the quantitative results reported above.

### 6.3. Limitations and directions for further research

Building on the above comparative discussion, this section outlines the main limitations of the present study and provides directions for further research.

#### 6.3.1. Conducting a comparative analysis of the Pearl growth curve to enhance the robustness of estimation results.

While this study demonstrates the advantages of the Gompertz specification, it also has several limitations that warrant further improvement.

This study, through normative analysis methods and comparison with the commonly used Pearl growth curve, finds that the Gompertz curve can describe the changes in the stage coefficient of cultivated land ecological value development from the perspective of policy supply, and is suitable for the development of ecological compensation policies. Consequently, the Gompertz curve was used to fit the development stage coefficient of cultivated land value. The analysis primarily focused on comparing the applicability of the two fitting methods through a normative approach, with a limited comparison of development stage coefficient values for 1984 and 2022 in Section 2.2. However, a comprehensive econometric comparison was not conducted.

Future work should not only compare coefficient values but also evaluate model fit using statistical criteria such as AIC, BIC, and RMSE across multiple datasets. Incorporating cross-validation and sensitivity analysis would further enhance the robustness of results and allow a more objective assessment of Pearl versus Gompertz performance.

#### 6.3.2. Expanding the study area to verify the generalizability of the model.

This study is based on a case study from Lezhi County. Although this region is somewhat representative, the generalizability of the research findings needs further verification. A broader research design should cover regions with different ecological functions, such as arid grasslands, forested watersheds, and peri-urban agricultural zones. Comparative studies across countries with varying institutional maturity would also test whether the Gompertz-based approach has global applicability.

#### 6.3.3. Optimizing the independent variable settings for the development stage coefficient to enhance the policy supply applicability and comprehensiveness of the model.

In this study, urbanization rate was selected as the independent variable for the development stage coefficient, as it effectively reflects the level of economic development and aligns with the orientation of ecological compensation policies. However, relying on a single indicator may not fully capture the comprehensive impacts of economic policies, market mechanisms, and land use planning on the EVCL, potentially limiting the model’s applicability and comprehensiveness in policy contexts.

Future models should test composite indices that integrate GDP per capita, fiscal transfers for ecological projects, and institutional capacity indicators. Such multidimensional measures would more fully reflect the drivers of ecological value change and enhance the policy relevance of the model.

### 6.4. Significance and contributions of the research

#### 6.4.1. Exploring the allocation of ecological value in cultivated land from a policy supply perspective: Expanding research methods with global applicability.

This study constructs a method for allocating the EVCL based on the perspective of ecological compensation policy supply, using the urbanization rate as the independent variable and the Gompertz curve for model fitting. Compared with the symmetric assumption underlying the Pearl growth curve, the Gompertz curve more accurately captures the asymmetric evolutionary process of ecological compensation policy (from initial slow development to rapid growth and eventual stabilization), thereby aligning with the practical logic of the progressive improvement and increased resource investment in ecological compensation policies. This method reflects the dynamic changes in the EVCL from the policy supply perspective, offering a new mathematical tool for research on ecological value allocation.

Traditional studies often rely on the Engel coefficient to measure the demand for ecological compensation; however, this indicator primarily reflects changes in household consumption structure and does not directly reflect the role of policy supply. In contrast, by selecting the urbanization rate as the independent variable, this study more directly illustrates the impact of urbanization on the evolution of ecological compensation policies. The results reveal that, with increasing urbanization, the supply of ecological compensation policies experiences a phased development process: beginning with initial institutional exploration, followed by rapid advancement, and finally reaching stabilization (a pattern that closely matches the growth characteristics of the Gompertz curve). This finding indicates that the supply of ecological compensation policies is not only driven by economic and social development but also exhibits distinct features of institutional evolution.

The method developed in this study is not only applicable to the analysis of ecological compensation policies in China but can also be extended to other countries and regions. In the context of accelerating global urbanization and significant changes in land use patterns, ecological compensation policies worldwide generally follow a similar trajectory of piloting, institutionalization, and continuous optimization. The ecological value allocation method based on the Gompertz curve proposed in this study thus provides a quantifiable analytical framework for the design of ecological compensation policies in different national contexts. It offers theoretical and methodological support for countries seeking to optimize their ecological compensation mechanisms according to their respective development stages, thereby enhancing the scientific rigor and operational effectiveness of policy implementation.

#### 6.4.2. Exploring the annual allocation approach of cultivated land ecological value, adapting to China’s ecological compensation value system.

This study proposes a method for allocating the EVCL based on fitting the development stage coefficient using the Gompertz curve, and subsequently calculates the annual EVCL. The results demonstrate that, with the progression of the development stage, the EVCL exhibits a steady growth trend. This trend reflects the positive impacts of China’s ecological civilization initiatives, the continuous improvement of ecological compensation mechanisms, and the implementation of sustainable land use policies on the enhancement of the ecological functions of cultivated land [63].

The dynamic assessment approach introduced in this study offers a feasible method for accurately capturing the long-term evolution of the EVCL. It provides scientific evidence to support the optimization of ecological compensation policy implementation in China. Policymakers can utilize the growth characteristics of ecological value across different regions to design more refined and stable ecological compensation mechanisms, thereby promoting the coordinated advancement of ecological conservation and economic development.

The research results effectively reflect the idea of internalizing ecological benefit externalities and the virtual marketization of ecological value, forming a positive feedback loop between ecological investment and ecological output. The annual ecological value estimates derived from this study can serve as the basis for developing an annual query table for ecological compensation values, providing technical support for the establishment of ecological compensation standards. This approach offers a valuable reference for policy initiatives such as the Grain for Green Program and watershed ecological compensation projects in China.

## 7. Research conclusions

### 7.1. Calculate the EVCL in Lezhi County

In this study, ecosystem service functions, including agricultural products and raw materials supply, water conservation, carbon sequestration and oxygen release, and soil and water conservation, were used to assess the EVCL (EVCL) in Lezhi County. The functional value method was applied to estimate the one-year value of each ecological service function. Based on this, the total EVCL in Lezhi County was calculated to be ¥53,055,425,310. In 2022, the total area of cultivated land in Lezhi County was 75,336.09 hectares. Using these figures, the EVCL per unit area was determined to be ¥704,250 per hectare per year.

### 7.2. Deriving the Gompertz curve equation through log-linearization and proxy variables for parameter estimation

This paper transforms the Gompertz curve model into a linear equation using the logarithmic linearization method. The urbanization rate is taken as the independent variable in the linear equation, while the GDP growth rate, which is highly correlated with the development stage coefficient, is selected as a proxy for the dependent variable in the linear equation. The linear equation is then fitted to obtain the parameters *a*, *b*, and *c*, which are used to derive the Gompertz curve equation $y=\text{2}\text{8}.\text{6}\text{4}e^{-\text{0}.\text{1}\text{2}\text{3}e^{-\text{0}.\text{0}\text{4}\text{5}x}}$.

### 7.3. Estimating the development stage coefficient based on the Gompertz curve equation and calculating the EVCL in Lezhi County

The gray prediction model GM(1,1) is employed to forecast the urbanization rate in Lezhi County from 2023 to 2052. Combined with historical data from 1979 to 2022, a complete time series is constructed and substituted into the Gompertz curve equation to obtain the corresponding curve values, by dividing by the saturation value of the Gompertz curve (*a* = 28.64), the development stage coefficient of the EVCL in Lezhi County from 1979 to 2052 is obtained. By multiplying the annual development stage coefficient by the per-hectare EVCL (¥704,250 per hectare per year), the EVCL in Lezhi County from 1979 to 2052 is derived.

### 7.4. Verifying the applicability of the Gompertz curve based on the S-shaped, asymmetric, and smooth evolution of the development stage coefficient

From 1979 to 2052, the development stage coefficient of the EVCL in Lezhi County increased from 0.0642 to 0.4545, showing a steady upward trend with an S-shaped and asymmetric evolution pattern. The growth was slow between 1979 and 1993, accelerated from 1994 to 2021, and began to decelerate after 2022, reflecting the staged and asymmetric nature of ecological value evolution. The fitted results align well with the theoretical form of the Gompertz curve, confirming its suitability and explanatory strength in simulating the long-term trajectory of ecological value.

Moreover, the Gompertz curve exhibits smooth-fitting characteristics and a self-correcting mechanism that reduces trend volatility and prevents over-adjustment, thereby enhancing model adaptability and stability. These results further support the applicability of the Gompertz curve in modeling the evolution of the EVCL.

This study contributes to the literature in three main aspects. First, it advances methodological research by introducing a Gompertz-based dynamic evaluation framework, which captures asymmetric growth processes that static and symmetric models fail to explain. Second, it provides empirical evidence from Lezhi County, a region with both ecological and institutional significance, thereby demonstrating how institutional supply interacts with ecological value evolution. Third, the findings extend policy relevance by offering a quantitative tool that links ecological value assessment with the design of compensation mechanisms, supporting more adaptive and stage-specific policy interventions.

Despite these contributions, limitations remain. The model was tested in one county only, and robustness should be examined across diverse regions with varying socio-economic and ecological conditions. In addition, future research could refine the independent variables by constructing a composite index that integrates economic, social, and institutional dimensions. Such improvements would enhance both the generalizability and the policy applicability of the framework.

Overall, this study not only quantifies the dynamic evolution of the EVCL but also provides a methodological reference for ecological value assessment and compensation policy design in other regions. By integrating theoretical innovation with policy application, the research underscores the role of dynamic modeling in advancing sustainable land management and ecological governance.

Future work should build upon the three directions outlined in the discussion section to further strengthen the model’s explanatory power and policy relevance. Specifically, conducting quantitative comparisons with the Pearl curve, expanding the analysis beyond a single county, and developing a composite index for the independent variable will enhance both the robustness and generalizability of the Gompertz-based framework.

By addressing these aspects, subsequent studies can refine the theoretical model and broaden its application in ecological compensation and sustainable land management across diverse socio-economic contexts.

## Supporting information

S1 Data Source data. (XLSX)

S2 Code Urbanization rate prediction code based on the GM(1,1) model. (DOCX)

S3 Data Urbanization rate and development stage coefficient of EVCL in Lezhi County from 1979 to 2052. (XLSX)

S4 Data EVCL in Lezhi County from 1979 to 2052. (XLSX)

S5 Appendix Simplified Pearl curve development stage coefficient calculation formula. (DOCX)
