# Supplementary material for: Dynamic assessment of the ecological value of cultivated land based on the Gompertz curve model: A case study of Lezhi County, China
Source: PLoS One. 2025 Dec 29;20(12):e0339281. doi: 10.1371/journal.pone.0339281 (PMC12747375; doi:10.1371/journal.pone.0339281)
Supplement: S2 Code — (DOCX) [file pone.0339281.s002.docx]

**S2 Code: Urbanization Rate Prediction Code Based on the GM(1,1) Model**

**# -*- coding: utf-8 -*-**

**"""**

Created on Fri Oct 10 19:24:49 2025

@author: university

**"""**

import numpy as np

import pandas as pd

**# Raw data**

years = list(range(1979, 2022))

urbanization_rate = [

7.59, 7.7, 8.14, 8.38, 8.52, 8.83, 9.93, 9.81, 9.82, 10,

10.05, 10.06, 10.19, 10.36, 10.92, 12.06, 12.28, 12.49, 12.87, 13.19,

14.03, 15.07, 15.98, 16.98, 18, 18.97, 19.11, 19.3, 20.9, 24.7,

27.5, 28.9, 29.2, 29.9, 30.8, 31.5, 32.4, 35.2, 36.4, 37.7,

39.2, 40.5, 41.32, 41.64

]

print(f"数据长度: {len(urbanization_rate)}")

**# Accumulative Generating Sequence (1-AGO)**

X0 = np.array(urbanization_rate, dtype=np.float64)

X1 = np.cumsum(X0)

print(f"X0: {X0}")

print(f"X1: {X1}")

**# Construct matrices Y and B**

n = len(X0)

Y = X0[1:].reshape(-1, 1)

**# Calculate the background value z^{(1)}(k)**

Z1 = np.zeros(n-1)

for i in range(1, n):

Z1[i-1] = 0.5 * (X1[i] + X1[i-1])

B = np.column_stack([-Z1, np.ones(n-1)])

print(f"Y shape: {Y.shape}")

print(f"B shape: {B.shape}")

**# Parameter Estimation - Using More Stable Computational Methods**

try:

**# Method 1: Use pseudo-inverse for greater stability**

a_b = np.linalg.pinv(B) @ Y

a = a_b[0, 0]

b = a_b[1, 0]

print(f" Parameter estimation successful: a = {a:.6f}, b = {b:.6f}")

except Exception as e:

print(f" Parameter estimation error: {e}")

**# Method 2: Alternative Calculation Method**

try:

a_b = np.linalg.inv(B.T @ B) @ B.T @ Y

a = a_b[0, 0]

b = a_b[1, 0]

print(f" Backup parameter estimation successful: a = {a:.6f}, b = {b:.6f}")

except Exception as e2:

print(f" Backup parameter estimation error: {e2}")

exit()

**# Validate parameters**

if abs(a) < 1e-10:

print("Warning: Parameter a is close to zero; the model may be unstable. ")

**# Cumulative Sequence Prediction Values**

def predict_X1(k):

return (X0[0] - b / a) * np.exp(-a * (k - 1)) + b / a

**# Predicted values for the original sequence**

def predict_X0(k):

if k == 1:

return X0[0]

return predict_X1(k) - predict_X1(k - 1)

**# Validate model fit**

print("\nModel Fitting Validation:")

for i in range(1, min(6, n+1)):

fitted = predict_X0(i)

actual = X0[i-1]

error = abs(fitted - actual)

print(f" year {i}: actual value = {actual:.2f}, fitted value = {fitted:.2f}, error = {error:.2f}")

**# Projected Urbanization Rate for 2023–2052**

future_years = list(range(2023, 2053))

future_predictions = []

print(f"\n start predicting {len(future_years)} year...")

for i, year in enumerate(future_years):

k = n + i + 1 # Start predicting from the 45th data point.

prediction = predict_X0(k)

future_predictions.append(prediction)

print(f" Predict the {k}th point (Year{year}): {prediction:.2f}")

**# Create the resulting DataFrame**

result = pd.DataFrame({

"Year": future_years,

" Urbanization Rate Forecast Results ": future_predictions

})

**# Set display options to show all rows**

pd.set_option('display.max_rows', None)

pd.set_option('display.float_format', '{:.2f}'.format)

print("\n" + "="*50)

print("Projected Urbanization Rates for 2023–2052:")

print("="*50)

print(result)

**# Save results to an Excel file**

try:

result.to_excel("Urbanization Rate Forecast Results _2023-2052.xlsx", index=False)

print("\n the forecast results have been saved to 'Urbanization Rate Forecast Results_2023-2052.xlsx'")

except Exception as e:

print(f"\n error occurred while saving the Excel file: {e}")

**# Display key statistics**

print("\n key statistics:")

print(f"development coefficient a: {a:.6f}")

print(f"gray effect b: {b:.6f}")

print(f"number of raw data points: {n}")

print(f"prediction range: 2023-2052 (for{len(future_years)}years)")
