# Supplementary material for: Dynamic assessment of the ecological value of cultivated land based on the Gompertz curve model: A case study of Lezhi County, China
Source: PLoS One. 2025 Dec 29;20(12):e0339281. doi: 10.1371/journal.pone.0339281 (PMC12747375; doi:10.1371/journal.pone.0339281)
Supplement: S5 Appendix — (DOCX) [file pone.0339281.s005.docx]

S5 Appendix:

Simplified Pearl curve developmental Stage coefficient calculation formula as follows:

where is the stage of development coefficient and $t$ is the inverse of the Engel coefficient.

In 1984, the Engel's coefficient of Lezhi County was 1.629, and the coefficient of the development stage was 0.8360; in 2022, the Engel's coefficient of Lezhi County was 2.674, and the coefficient of the development stage was 0.9355.
